# ERα inhibits epithelial-mesenchymal transition by suppressing Bmi1 in breast cancer

**DOI:** 10.18632/oncotarget.3966

**Published:** 2015-05-13

**Authors:** Xiao-Long Wei, Xiao-Wei Dou, Jing-Wen Bai, Xiang-Rong Luo, Si-Qi Qiu, Di-Di Xi, Wen-He Huang, Cai-Wen Du, Kwan Man, Guo-Jun Zhang

**Affiliations:** ^1^ Department of Pathology, Cancer Hospital of Shantou University Medical College, Shantou 515031, China; ^2^ Changjiang Scholar's Laboratory and Cancer Research Center, Shantou University Medical College, Shantou 515031, China; ^3^ The Breast Center, Cancer Hospital of Shantou University Medical College, Shantou 515031, China; ^4^ Department of Breast Medical Oncology, Cancer Hospital of Shantou University Medical College, Shantou 515031, China; ^5^ Department of Surgery and Transplantation, Li Ka Shing Faculty of Medicine, Hong Kong University, Hong Kong 999077, China

**Keywords:** ERα signaling, epithelial-mesenchymal transition, Bmi1, breast cancer, stemness

## Abstract

In human breast cancer, estrogen receptor-α (ERα) suppresses epithelial-mesenchymal transition (EMT) and stemness, two crucial parameters for tumor metastasis; however, the underlying mechanism by which ERα regulates these two processes remains largely unknown. Bmi1, the polycomb group protein B lymphoma Mo-MLV insertion region 1 homolog, regulates EMT transition, maintains the self-renewal capacity of stem cells, and is frequently overexpressed in human cancers. In the present study, ERα upregulated the expression of the epithelial marker, E-cadherin, in breast cancer cells through the transcriptional down-regulation of Bmi1. Furthermore, ERα overexpression suppressed the migration, invasion, and EMT of breast cancer cells. Notably, overexpression of ERα significantly decreased the CD44^high^/CD24^low^ cell population and inhibited the capacity for mammosphere formation in ERα-negative breast cancer cells. In addition, overexpression of Bmi1 attenuated the ERα-mediated suppression of EMT and cell stemness. Immunohistochemistry revealed an inverse association of ERα and Bmi1 expression in human breast cancer tissue. Taken together, our findings suggest that ERα inhibits EMT and stemness through the downregulation of Bmi1.

## INTRODUCTION

Of all cancers, that of the breast is the most common for women, with most breast cancer-related deaths involving widespread metastasis [1]. Estrogen receptor-α (ERα) is a nuclear receptor that is activated by the sex hormone, estrogen; it regulates the transcription of estrogen-responsive genes in diverse target cells. Ligand binding induces a conformational change within ERα, thus promoting dimerization and high-affinity binding to specific estrogen-responsive elements (EREs) located within the promoter of target genes [2]. ERα is an important prognostic indicator in breast cancer [3]. ERα signaling promotes the growth of primary breast cancers, but can also antagonize signaling pathways that lead to epithelial-mesenchymal transition (EMT) [1, 2, 4–7]. ERα-positive breast cancer cells appear to contain a relatively small subpopulation of breast cancer stem cells [8, 9]. Post-EMT breast cancer cells express cancer stem cell markers, including Bmi1, but show decreased ERα expression [10, 11]. However, the mechanisms by which ERα regulates EMT, as well as inhibits stemness in breast cancer, remain to be explored.

In EMT, epithelial cells lose their polarity and acquire the migratory and invasive properties of mesenchymal cells. Cell-to-cell adhesion is mediated by cadherins such as E-cadherin, which provides a structural support for cell-cell attachment. Thus, a loss of E-cadherin expression can cause a loss of polarity of epithelial cells and is considered a fundamental event in EMT [12, 13]. In addition to its crucial role in the differentiation of many tissues and organs, EMT has also been shown to cause organ fibrosis and promote carcinoma progression through a variety of mechanisms [13]. Recent reports suggest a direct link between EMT and the gain of stem-like properties [12, 13]. Induction of EMT not only allows cancer cells to disseminate from the primary tumor, but also promotes their self-renewal capability. In breast cancer, EMT is associated with cancer stem cell properties, including the expression of a stem cell-associated CD44^high^/CD24^low^ antigenic profile, and self-renewal capabilities [10]. Despite this, the molecular pathways linking EMT to the acquisition of stem cell properties remain, as yet, largely undefined.

Increasing evidence suggests that the polycomb group of transcription factor proteins plays a crucial role in cancer development and recurrence. Of these, Bmi1 is a member of the polycomb-repressive complex 1, which is strongly involved in the self-renewal of stem cells and is associated with a number of human malignancies such as oropharyngeal cancer, neuroblastoma and melanoma [14–18]. Bmi1 maintains the self-renewal of both normal and malignant human mammary stem cells [19], suppresses E-cadherin, and enhances stemness in head and neck cancer cells [20]. Bmi1 also plays an important role in both EMT and stemness processes in human nasopharyngeal and pancreatic cancers [21, 22]. In a previous study, ERα was demonstrated to up-regulate the expression of E-cadherin both directly and indirectly [2, 7, 23, 24]. However, whether EMT inhibition by ERα is mediated through a direct/indirect upregulation of E-cadherin remains poorly understood, and further, the regulatory mechanism of Bmi1 in cancer cells and its role in metastasis are largely unknown.

We hypothesized that ERα may upregulate E-cadherin through a Bmi1-mediated pathway. We investigated this process *in vitro* in breast cancer cells, and *in vivo* in a mouse model and human breast cancer tissues.

## RESULTS

### Estrogen hormone (E2) and ERα downregulates Bmi1 expression and increases E-cadherin expression in breast cancer cells

**Figure 1:**
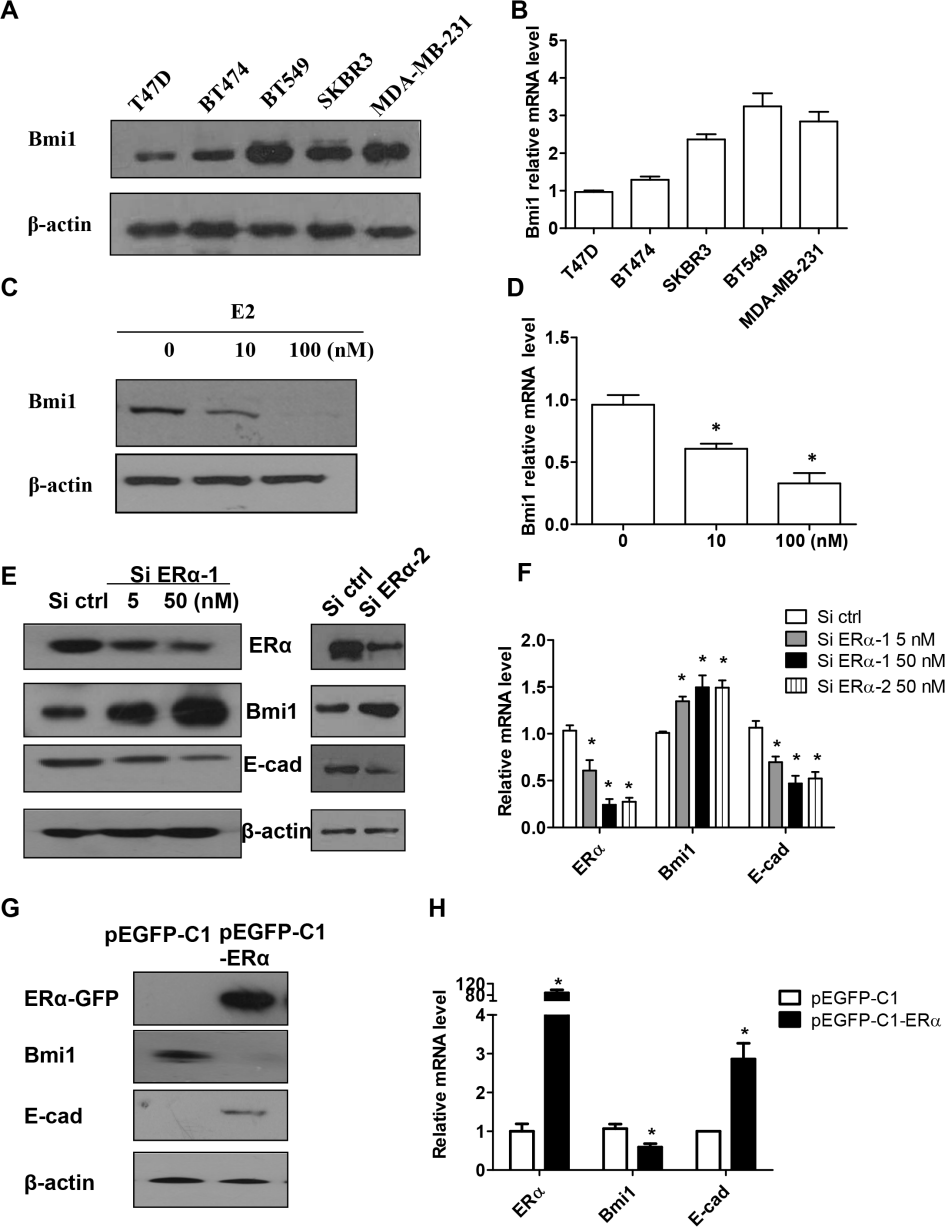
E2 and ERα downregulates Bmi1 expression and increases E-cadherin expression in breast cancer cells **A–B.** Western blot (A) and quantitative real-time RT-PCR analysis (B) of protein and mRNA expression of Bmi1 in ERα-positive T47D and BT474 cells, and ERα-negative breast cancer cells (SKBR3, BT549 and MDA-MB-231). **C–D.** T47D cells were maintained in phenol red-free DMEM with 10% dextran-coated charcoal-treated FBS for 48 h, and cells then treated with either ethanol vehicle or E2 (10 or 100 nM) for 72 h. Cells were harvested and analyzed for Bmi1 mRNA and protein levels. Data are mean ± SEM (*n* = 3). *P < 0.05 compared with ethanol vehicle (Student's *t* test). **E–F.** Western blot (E) and quantitative RT-PCR analysis (F) of protein and mRNA levels in T47D cells transfected with control siRNA, ERα siRNA-1 (5 and 50 nM) or ERα siRNA-2 (50 nM). **G–H.** ERα, Bmi1 and E-cadherin protein and mRNA levels in BT549 cells stably transfected with the indicated vectors. Protein expression was normalized to β-actin mRNA. *P < 0.05 compared with control cells (Student's *t* test).

As was previously reported, post-EMT breast cancer cells express cancer stem cell markers, including Bmi1, but display decreased ERα expression [1]. In order to quantify Bmi1 expression in breast cancer cells, we detected Bmi1 protein expression by Western blot in various breast cancer cell lines. We found that Bmi1 expression was higher in three ERα-negative breast cancer cell lines (SKBR3, BT549, and MDA-MB-231) than in ERα-positive T47D or BT474 cells (Figure 1A). To compare Bmi1 mRNA expression in these lines, real-time RT-PCR was performed, with β-actin used as an internal control. Consistent with Bmi1 protein expression, Bmi1 mRNA levels were 2 to 3 fold higher in ERα-negative breast cancer cell lines than in in ERα-positive cells (Figure 1B).

Because both protein and mRNA levels of Bmi1 were decreased in ERα-positive T47D cells relative to ERα-negative breast cancer cell lines, we determined whether ERα signaling played a role in Bmi1 expression. T47D cells cultured in estrogen-depleted medium were treated with various concentrations of E2. After 24 or 72 h, Bmi1 protein and mRNA levels were dose-dependently downregulated by E2 (Supplemental Figures 1A, 1B; Figures 1C, 1D, respectively). When the cells were treated with E2 at 10^−7^ M for 72 h, Bmi1 mRNA levels were significantly reduced by approximately 70% (Figure 1D; *P* < 0.05), and protein levels were decreased by more than 90% (Figure 1C).

To further investigate the impact of ERα on Bmi1, we silenced endogenous ERα in T47D cells using siRNA and examined Bmi1 and E-cadherin expression. As shown in Figures 1E and 1F, silencing endogenous ERα in T47D cells led to the significant up-regulation of Bmi1 and significant down-regulation of E-cadherin at both protein and mRNA levels (*P* < 0.05), respectively, in a dose-dependent manner.

To investigate the effect of ERα on Bmi1 expression in an ERα-negative breast cancer cell line, we stably transfected the recombinant vector pEGFP-C1-ERα, or an empty vector, into ERα-negative BT549 cells. ERα protein and mRNA levels (*P* < 0.05) were increased in pEGFP-C1-ERα-, but not control, vector-transfected BT549 cells (Figures 1G and 1H, respectively). We further analyzed the expression of Bmi1 and E-cadherin. Bmi1 was markedly downregulated and E-cadherin was upregulated at both the protein level, and significantly, at the mRNA level in pEGFP-C1-ERα, as compared with pEGFP-C1 transfected BT549 cells (Figures 1G, 1H; *P* < 0.05 for mRNA).

### ERα down-regulates Bmi1 expression by directly binding to the *BMI1* promoter

**Figure 2:**
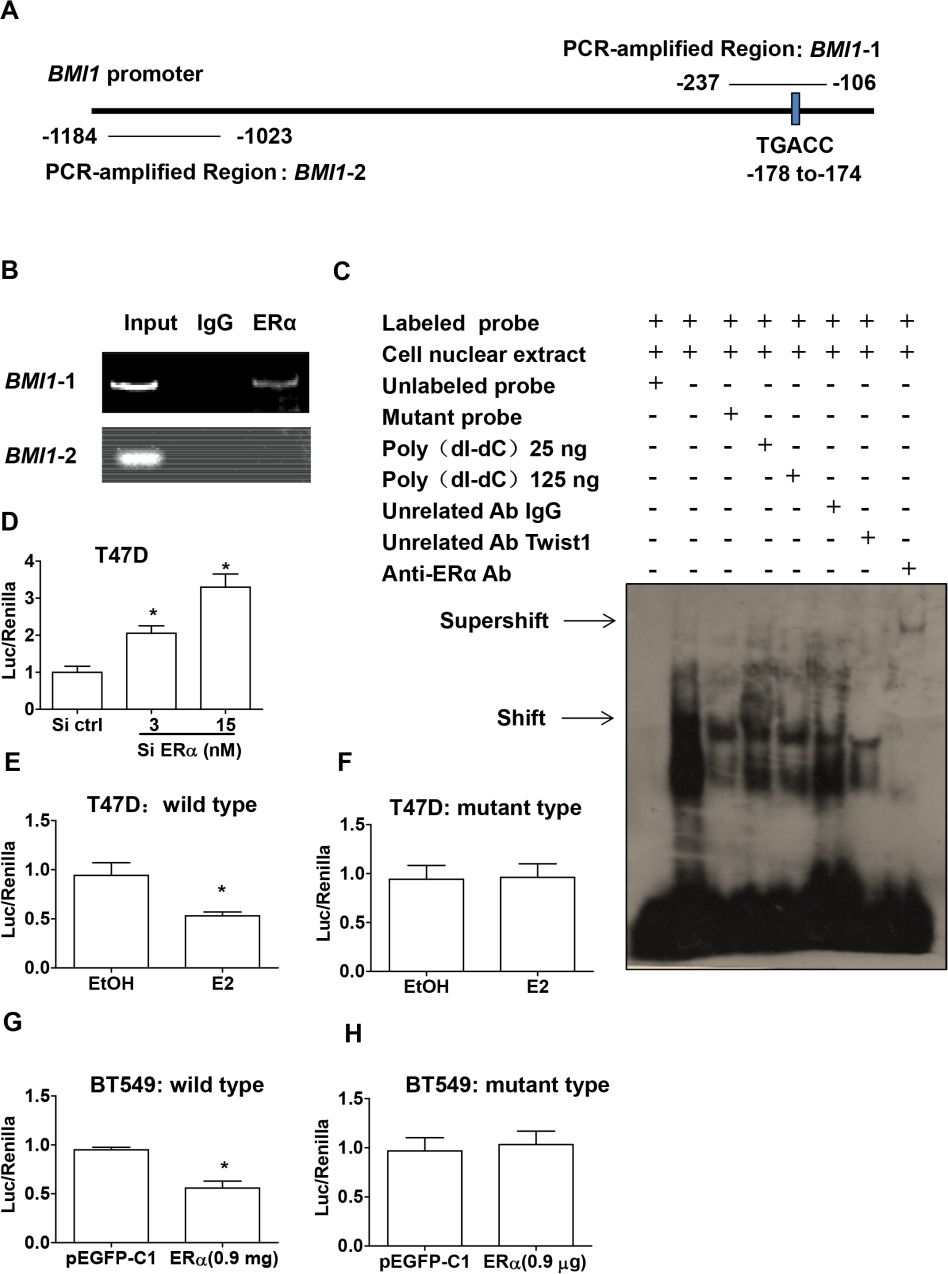
Bmi1 expression is directly regulated by ERα **A.** A schematic representation of the *BMI1* promoter region, with or without the half-ERE site for ERα. Precipitated DNA was amplified by PCR with specific primers for regions 1–2. **B.** Chromatin immunoprecipitation (ChIP) assay involved normal IgG (IgG), or anti-ERα (ERα) antibody to identify the ERα binding site on the *BMI1* promoter in T47D cells. **C.** Electrophoretic mobility shift assay (EMSA). Nuclear extracts from pEGFP-C1-ERα-transfected BT549 cells were incubated with a probe labeled at its 3′-end with biotin from the *BMI1* promoter region containing the ERα binding site (lanes 1–8). Unlabeled probe (lane 1) or mutant oligonucleotides (lane 3) were added at 50-fold greater concentrations than that of the labeled probe. In the non-specific binding assay, 25 or 125 ng of poly (dI: dC) (lanes 4–5), normal IgG (lane 6), or anti-Twist1 antibody (lane 7) was added. For the supershift assay, the addition of anti-ERα antibody resulted in a supershifted band (lane 8). **D.** T47D cells were co-transfected with a *BMI1* promoter construct (pXP2-BMI1-Luc1000) and ERα siRNA (3 or 15 nM). **E.** T47D cells were transfected with a *BMI1* promoter construct (pXP2-BMI1-Luc1000) and treated with either ethanol vehicle (EtOH) or 100 nM E2 (E2) for 24 h. **F.** T47D cells were transfected with pXP2-BMI1-mutant and treated with either ethanol vehicle (EtOH) or 100 nM E2 (E2) for 24 h. **G.** BT549 cells were co-transfected with pXP2-BMI1-Luc1000 and either pEGFP-C1 or pEGFP-C1-ERα. **H.** BT549 cells were co-transfected with pXP2-BMI1-mutant and either pEGFP-C1 or pEGFP-C1-ERα. E–H, All cells were co-transfected with Renilla luciferase plasmid. Luciferase activity was normalized to that of Renilla. *P < 0.05 (Student's *t* test) compared with control cells. Data are mean ± SEM (*n* = 3).

Based on our previous findings that Bmi1 expression is transcriptionally regulated by ERα signaling, we addressed whether ERα can directly bind to the regulatory regions of the *BMI1* promoter. To determine the binding site, we searched for specific EREs located within the *BMI1* promoter. We did not find classical ERE sites but instead found a half-ERE site at position −178 to −174 (Figure 2A). To investigate whether ERα could form a complex with the *BMI1* promoter, we performed a chromatin immunoprecipitation (ChIP) assay with primers covering the *BMI1* promoter region. We used a far upstream region, beyond the half-ERE site, in the *BMI1* promoter as a negative control. ERα bound to the region between positions −237 to −106 containing the half-ERE site, but not the region between positions −1184 to −1023, which did not contain the ERE/half-ERE binding site (Figure 2B).

Electrophoretic mobility shift assay (EMSA) revealed an ERα-binding band after the incubation of nuclear extracts from BT549 cells overexpressing ERα with labeled oligonucleotides containing the half-ERE from the *BMI1* regulatory region; a supershifted band was seen after the addition of an anti-ERα-specific antibody to the mixture (Figure 2C). The addition of excess amounts of unlabeled oligonucleotides containing the half-ERE outcompeted and abolished the binding activity of ERα, but not the addition of mutant half-ERE-containing unlabeled oligonucleotides or non-specific competition by poly (dI:dC). No supershifted band was seen after the addition of an unrelated antibody, normal IgG, or anti-Twist1 antibody, which binds to the E box of the *BMI1* promoter.

To determine the direct regulation of *BMI1* by ERα, we used a luciferase reporter vector that included the region from −1000 to −1 of the *BMI1* gene. A dual luciferase reporter assay was used to investigate *BMI1* promoter activity in ERα-silenced T47D cells or ERα-overexpressing BT549 cells by co-transfection with the *BMI1* promoter and Renilla luciferase reporter genes. Transfection with ERα siRNA significantly increased *BMI1* promoter activity 2- to 3.5-fold in T47D cells in a dose-dependent manner (Figure 2D; *P* < 0.05). E2 administration decreased wild-type *BMI1* promoter activity to 0.5-fold (Figure 2E; *P* < 0.05) but did not show any effect on the mutant *BMI1* promoter (Figure 2F). Similarly, ectopic ERα overexpression significantly decreased *BMI1* promoter activity to 0.5-fold in BT549 cells (Figure 2G; *P* < 0.05), but showed no effect on mutant *BMI1* promoter activity (Figure 2H). These results demonstrated that ERα directly repressed *BMI1* transcription by specifically binding to the promoter region of the *BMI1* gene.

### ERα suppresses EMT and stemness in breast cancer cells

**Figure 3:**
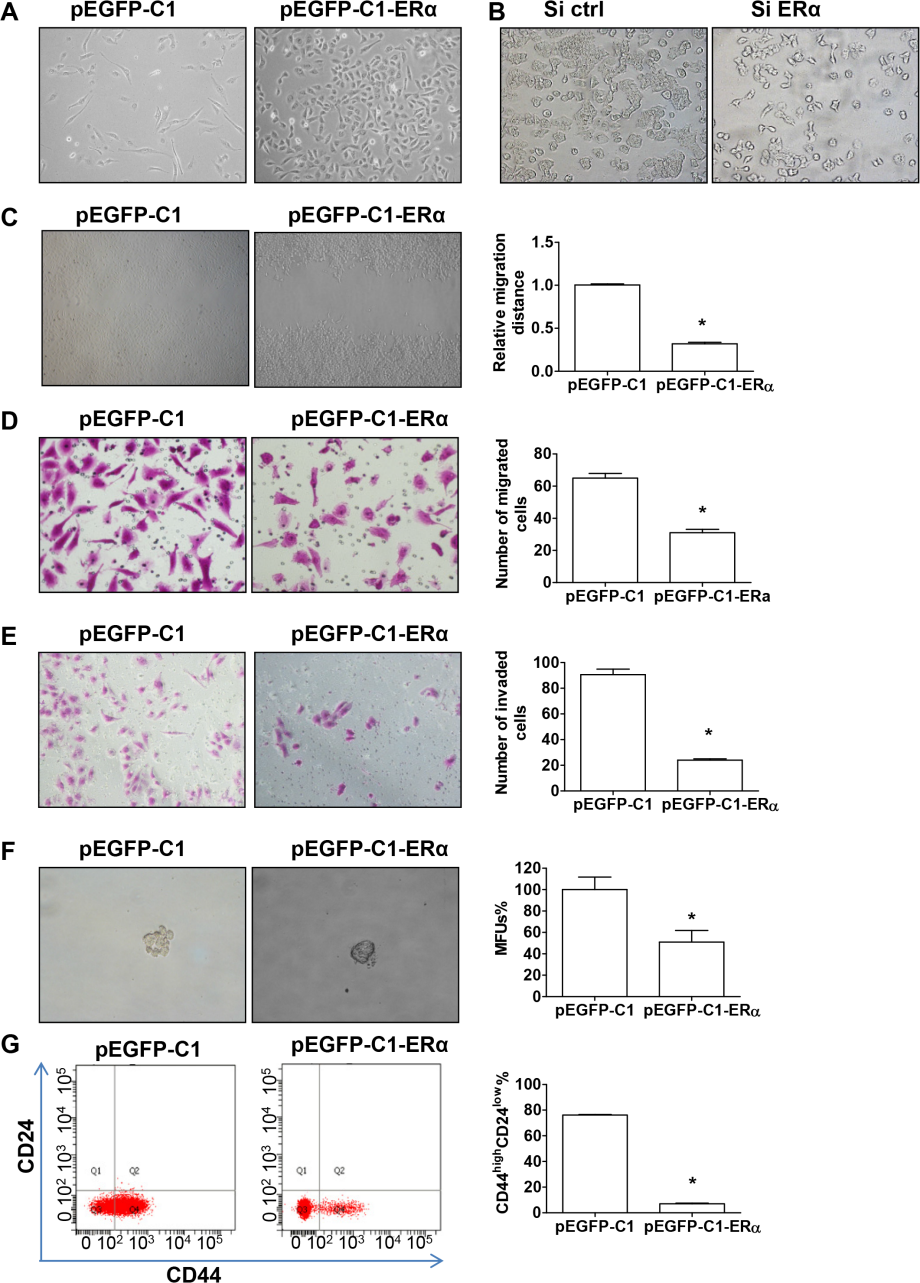
ERα suppresses EMT and cell stemness **A.** Morphology of pEGFP-C1 and pEGFP-C1-ERα BT549 cells. **B.** Morphology of T47D cells transfected with control or ERα siRNA. **C.** Wound healing assay in pEGFP-C1 or pEGFP-C1-ERα BT549 cells. **D–E.** Migration and invasion assays of pEGFP-C1 and pEGFP-C1-ERα BT549 cells. Representative images of migrating (D) or invading (E) cells are shown. **F.** Representative images of mammosphere forming units (MFUs) observed in pEGFP-C1 and pEGFP-C1-ERα BT549 cells. Data are mean ± SEM (*n* = 3). **G.** FACS analysis of cell-surface markers CD44 and CD24 in pEGFP-C1 and pEGFP-C1-ERα BT549 cells. Data are mean ± SEM (*n* = 3). *P < 0.05 compared to pEGFP-C1 BT549 cells (Student's *t* test). Magnification, 400 × (A–B, D–F); 100 × (C).

We next examined BT549 cells, stably transfected with either pEGFP-C1 or pEGFP-C1-ERα vector, for morphological changes indicating EMT (Figure 3A). pEGFP-C1 BT549 cells possessed a fibroblast-like morphology and displayed pronounced cellular scattering, while pEGFP-C1-ERα BT549 cells expressing ERα showed a morphological conversion from a spindle-shaped, mesenchymal morphology to a cuboidal, epithelial morphology (Figure 3A). Through seeding small amounts of cells, apparent morphological change could be seen after 48 h culture (Supplemental Figure 2). In contrast, silencing endogenous ERα expression in T47D cells resulted in a partial loss of epithelial characteristics (Figure 3B).

To further investigate the impact of ERα on EMT, we determined migratory and invasive behaviors in ERα stably-transfected BT549 cells. The healing of wounded areas was significantly slower by about 65% in pEGFP-C1-ERα, compared to pEGFP-C1, BT549 cells (Figure 3C; *P* < 0.05). Overexpression of ERα in BT549 cells significantly decreased the level of migration to about 50% of the control (Figure 3D; *P* < 0.05). Matrigel invasion chamber assays revealed that overexpression of ERα markedly reduced, to about 35% of capacity, the invasiveness of BT549 cells (Figure 3E: *P* < 0.05).

Post-EMT mammary epithelial cells show an increased capability of mammosphere formation and an increased CD44^high^/CD24^low^ cell population, both of which are characteristics of normal mammary and breast cancer stem cells [14–16]. The mammosphere formation assay showed a significant decrease in both the size and number of mammospheres in pEGFP-C1-ERα BT549 cells (Figure 3F; *P* < 0.05 and Supplemental Figure 3). To determine whether ERα expression decreased the cancer stem cell-like cell population by suppressing EMT, we analyzed the CD44^high^/CD24^low^ cell population in ERα stably-transfected BT549 cells. Most of the control pEGFP-C1 BT549 cells showed a CD44^high^/CD24^low^ phenotype, which was significantly decreased, from 76% to 7% of the cell population in pEGFP-C1-ERα BT549 cells (Figure 3G; *P* < 0.05). These results indicate that ERα suppresses both the expression of cell surface markers and functional characteristics associated with cancer stem cells.

### ERα decreased the metastatic ability of BT549 cells *in vivo*

**Figure 4:**
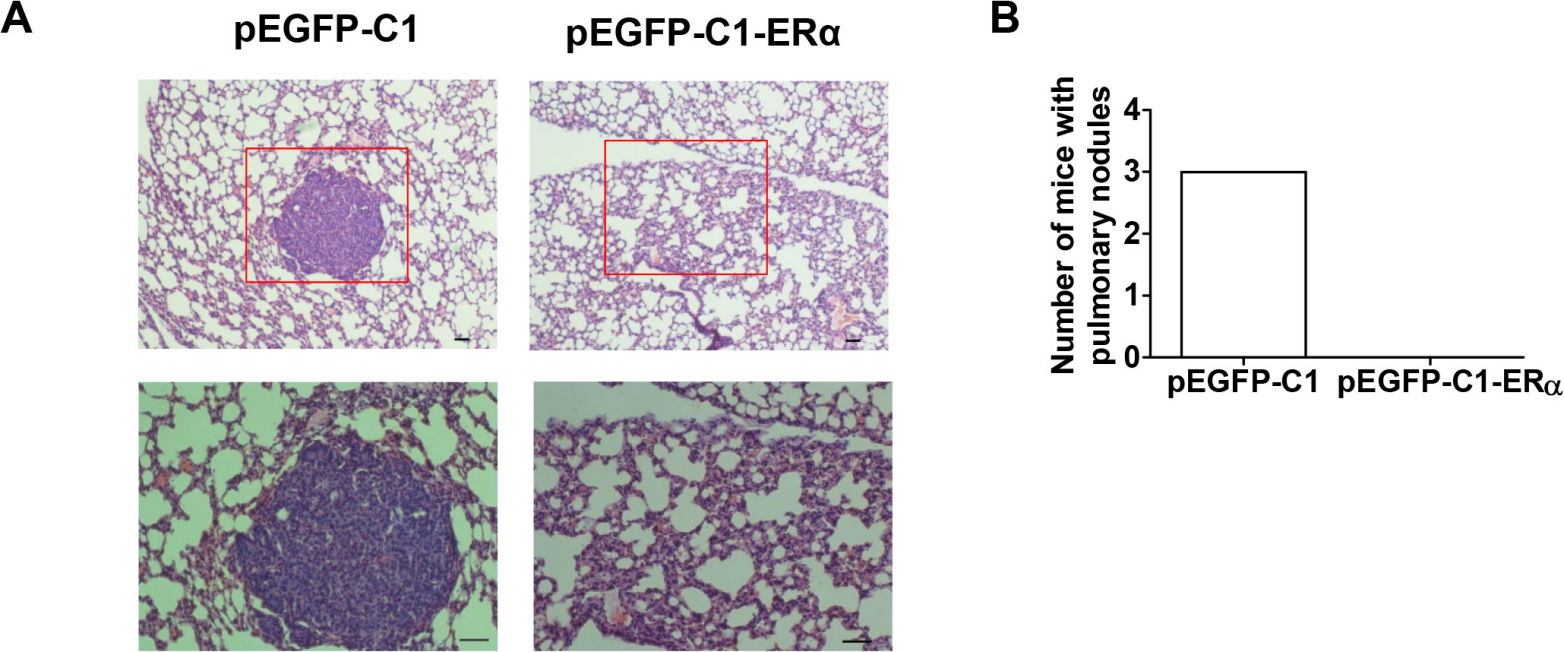
ERα expression reduces the metastasis of BT549 cells **A.** H&E staining of metastatic nodules (red box) in mouse lung tissue. Magnification, 100× (upper); 200× (bottom). Bar = 50 μM. **B.** Number of mice with pulmonary nodules (*n* = 5).

To test whether ERα overexpression indeed decreases the metastatic ability of BT549 cells *in vivo*, a tail vein metastatic model was established in BALB/c nude mice. At 6 weeks following the injection of tumor cells, animals were sacrificed and lung metastasis was examined by H&E staining. Three of the five mice implanted with BT549 cells developed lung metastases. In contrast, mice implanted with BT549 cells overexpressing ERα showed no evident metastatic nodules in lungs (Figures 4A, 4B). Experimental and control groups did not show metastasis in the liver (data not shown).

### Restoration of Bmi1 expression reverses ERα-mediated suppression of EMT and stemness

**Figure 5:**
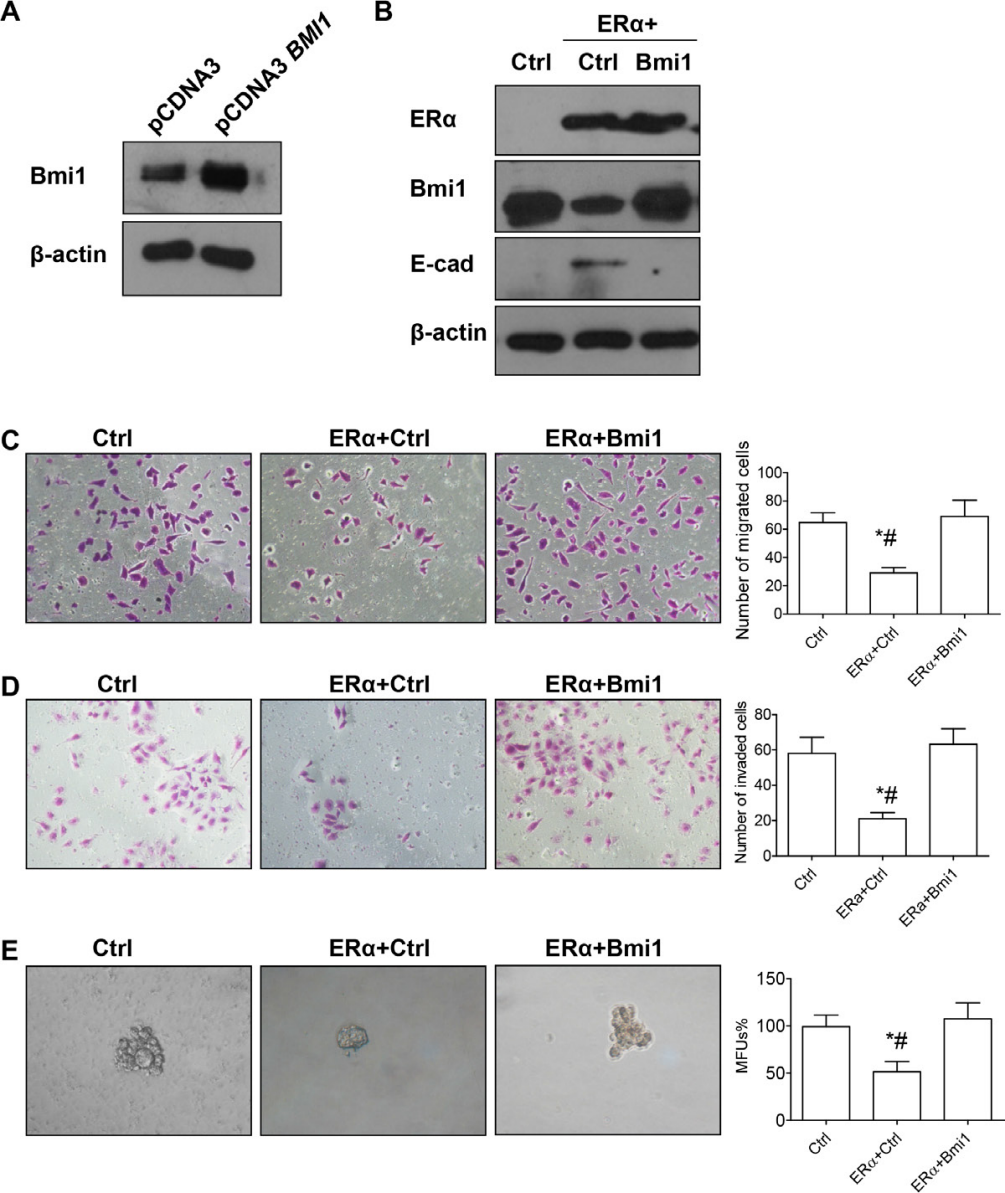
Restoration of Bmi1 expression reverts ERα-suppressed EMT and stemness **A.** Western blot analysis of Bmi1 protein in BT549 cells transfected with the indicated vectors. **B.** Western blot analysis of ERα, Bmi1 and E-cadherin protein levels in pEGFP-C1 or pEGFP-C1-ERα BT549 cells transiently transfected with the indicated vectors. **C–D.** Migration and invasion assays. Representative images of migrating (C) or invading cells (D) are shown. **E.** Morphology and quantification of mammospheres. Ctrl: pEGFP-C1 BT549 cells transiently transfected with control plasmid pcDNA3; ERα+Ctrl: pEGFP-C1-ERα BT549 cells transiently transfected with pcDNA3; ERα+Bmi1: pEGFP-C1-ERα BT549 cells transiently transfected with pcDNA3-BMI1. *P < 0.05, pEGFP- C1-ERα BT549 cells transfected with pcDNA3 (ERα+Ctrl) compared with pEGFP-C1 BT549 cells transfected with pcDNA3 (Ctrl). #*P* < 0.05, pEGFP-C1-ERα BT549 cells transfected with pcDNA3 (ERα+Ctrl) compared with pEGFP-C1-ERα BT549 cells transfected with pcDNA3 BMI1 (ERα+Bmi1) (Student's *t* test). Data are mean ± SEM (*n* = 3). Magnification, 400× (C–E).

Next, we investigated the role of Bmi1 in ERα-mediated suppression of EMT and stemness properties in breast cancer cells. A Bmi1 expression vector or empty vector was transiently transfected into pEGFP-C1-ERα BT549 cells; restoration of Bmi1 expression resulted in the loss of E-cadherin expression (Figures 5A, 5B), significantly increased migration or invasion (Figures 5C, 5D; *P* < 0.05 for both), and significantly increased mammosphere-forming ability (Figure 5E; *P* < 0.05). Restoration of Bmi1 expression did not affect the protein level of ERα (Figure 5B), and we therefore conclude that ERα is an upstream regulator of Bmi1, with no feedback regulation between ERα and Bmi1.

### Bmi1 expression is negatively associated with ERα and E-cadherin levels in human breast cancer

**Figure 6:**
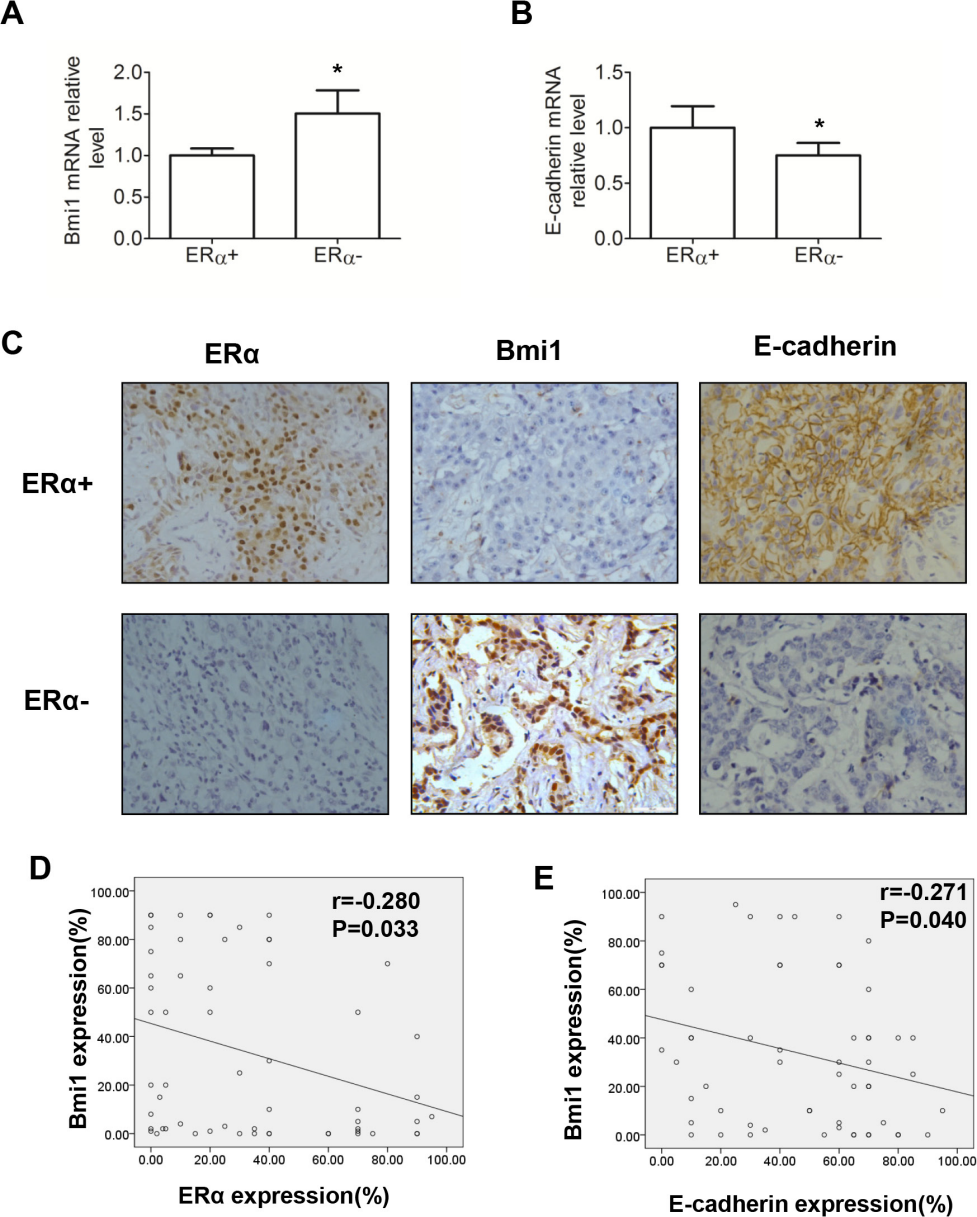
Association of Bmi1 and E-cadherin expression in 58 human breast cancer tissues **A, B.** Relative mRNA levels of Bmi1 (A) and E-cadherin (B) with ERα-negative and ERα-positive expression in breast cancer tissues. *P < 0.05 (Student's *t* test). **C.** Representative fields of positive ERα, negative Bmi1 and positive E-cadherin (upper row) and negative ERα, positive Bmi1, and negative E-cadherin (lower row). Magnification, 400× (C) **D.** Scatter plot of Bmi1 and ERα positive, immunohistochemically stained percentages of cells in human breast cancer tissues. **E.** Scatter plot of Bmi1 and E-cadherin positive, immunohistochemically stained percentages of cells in human breast cancer tissues (Spearman's Rank Correlation Test).

We further analyzed the mRNA levels of Bmi1 and E-cadherin in 58 human breast cancer samples. Bmi1 mRNA levels were significantly lower in ERα-positive than in ERα-negative tissues (Figure 6A; *P* < 0.05), but E-cadherin mRNA levels were significantly higher in ERα-positive than in ERα-negative tissues (Figure 6B; *P* < 0.05). Furthermore, immunohistochemistry staining for Bmi1, ERα and E-cadherin revealed that Bmi1 expression was inversely associated with ERα and E-cadherin levels in human breast cancer specimens in a significant manner (Table 1 and Figure 6C; *P* < 0.05). We demonstrated an inverse linear correlation of ERα or E-cadherin expression, with Bmi1 expression, when the percentage of positively stained cells for each protein was determined in human breast cancer tissues (Figures 6D and 6E; *P* < 0.05). These results support a model of ERα interacting with the Bmi1 locus, leading to the transcriptional repression of Bmi1 for the downregulation of E-cadherin.

**Table 1:** Association of Bmi1, ERα and E-cadherin expression in human breast cancer tissues

	*n*	Bmi1		*P* value
		Negative (%)	Positive (%)	
ERα				
Negative	26	13 (50.0)	13 (50.0)	0.003
Positive	32	28 (87.5)	4 (12.5)	
E-cadherin				
Negative	28	16 (57.1)	12 (42.9)	0.043
Positive	30	25 (83.3)	5 (16.7)	

## DISCUSSION

Breast cancer is the most commonly diagnosed cancer in women and remains one of the leading causes of death [1]. Most breast cancer-related deaths occur after extensive metastasis, during which EMT endows cancer cells with increased migration and invasive capabilities. Since ERα has been shown to suppress EMT, efforts to elucidate the molecular mechanisms by which ERα regulates EMT, as well as inhibits stemness, in breast cancer are warranted in order to develop novel treatments for this common type of cancer. In the current study, we have demonstrated that ERα can suppress Bmi1 expression by transcriptional repression, and inhibit EMT and stemness in breast cancer through an ERα-Bmi1-E-cadherin pathway.

The loss of E-cadherin expression is considered to be a fundamental event in EMT [13]. Several mechanisms, including transcriptional repression and methylation of the promoter, have been shown to repress E-cadherin expression. Many transcription factors, such as Snail, Slug and Zeb1, can bind to the E-cadherin promoter and directly repress its transcription [25–28], whereas other factors, such as Twist1 and FoxC2, can repress E-cadherin indirectly. This results in a disrupted polarity of epithelial cells and induced EMT [29, 30], thus maintaining the mesenchymal phenotype and enhancing the invasiveness and metastasis of cancer cells [31].

Bmi1 can downregulate E-cadherin expression and induce EMT in head and neck cancer by directly binding to E-box consensus sequences in the promoter region of E-cadherin [20]. Bmi1 can also indirectly repress E-cadherin expression by activating Snail in breast [32] and nasopharyngeal cancer [21]. In breast cancer, Bmi1 and E-cadherin levels were inversely related, while Bmi1 expression inversely correlated with the prognosis [32]. Although numerous studies have demonstrated that the regulation of E-cadherin can be mediated by ERα, and that the loss of E-cadherin was regulated by Bmi1, no studies have outlined the involvement of Bmi1 in an ERα-mediated E-cadherin regulation and EMT transition.

The presence of ERα represents a epithelial phenotype in the breast. Previous studies suggested that ERα upregulates several epithelial markers, such as E-cadherin, in breast cancer cells. In studying the role of Bmi1 in hormone-dependent and -independent breast cancer cells, we found that Bmi1 mRNA and protein levels were lower in ERα-positive than in ERα-negative cells. Furthermore, estrogen stimulation downregulated Bmi1 expression in hormone-dependent breast cancer cells, while in hormone-independent cells, Bmi1 was downregulated by ERα overexpression. When ERα expression was silenced by RNA interference, Bmi1 became upregulated. These findings suggest that ERα modulates the Bmi1 signaling pathway.

ERα has previously been shown to bind to the ERE/half-ERE region in the promoter of its downstream genes to regulate gene expression [33, 34]. For instance, ERα can repress Slug transcription by binding to the half-ERE element in the Slug promoter to regulate E-cadherin expression and EMT [2, 7]. Interestingly, the *BMI1* promoter sequence includes a half-site ERE element (TGACC). Evidence that ERα directly binds to the TGACC site in the *BMI1* promoter suggests that ERα-mediated Bmi1 suppression is indeed regulated at the transcriptional level. This conclusion is further supported by our dual luciferase reporter gene assay revealing increased/decreased activity of a *BMI1* promoter-driven reporter gene in ERα silenced/overexpressing breast cancer cells. Our findings indicate that ERα suppresses Bmi1 expression by directly binding to the half-ERE site of the *BMI1* promoter, thereby demonstrating a novel pathway by which ERα suppresses migration, invasion, and EMT in breast cancer cells, in addition to modulating Slug [2, 7] and NF-κB [6] pathways. Interestingly, a recent article by Wang *et al*. reported that ERα bound to the promoter of Bmi1, as also identified by our study, suggesting that the ERα could transcriptionally modulate Bmi1 expression [35]. In contrast to our findings, however, Wang's study found that Bmi1 expression was upregulated by the overexpression of ERα in another ERα-positive breast cancer cell line, MCF-7, while depletion of this protein caused a down-regulation of Bmi1. Whether this well-demonstrated, opposing regulation is cell line-specific needs to be investigated in future studies.

Song *et al*. showed that Bmi1 could inhibit phosphatase and tensin homologs, induce EMT and also regulate the self-renewal and differentiation of stem cells in human nasopharyngeal epithelial cells [21]. In pancreatic cancer, the EMT-activator, Zeb1, maintains stemness of cells, in part, through Bmi1 [22]. In our study, the elevated expression of ERα decreased the CD44^high^/CD24^low^ cell population in breast cancer cells and inhibited the cells’ capacity to form mammospheres. Most importantly, restoring Bmi1 expression reversed the ERα-mediated suppression of EMT and stemness. The current findings highlight the critical role of Bmi1 in regulating both EMT and stemness in breast cancer cells. Although ERα expression represses the stemness of cancer cells, the underlying mechanism of ERα in regulating stemness has not been thoroughly explored. ERα may modulate the stemness of breast cancer cells by suppressing Bmi1 expression, and may therefore be considered an inhibitor of circulating and migrating cancer stem cells.

In summary, our results demonstrate that ERα can suppress EMT in human breast cancer cells through the transcriptional down-regulation of Bmi1 and its down-stream genes. An inverse relationship between ERα and Bmi1 expression further supports the epithelial phenotype of ERα-positive tumors, or the mesenchymal phenotype of ERα-negative tumors, as being most likely regulated via the ERα-Bmi1-E-cadherin axis. Our findings provide a novel mechanistic insight into how ERα regulates EMT and may be of value in developing new biomarkers for the prognosis of breast cancer.

## MATERIALS AND METHODS

### Cell lines, antibodies and plasmids

BT474, T47D, MDA-MB-231, BT549 and SKBR3 breast cancer cell lines were purchased from the Committee on Type Culture Collection of the Chinese Academy of Science (Shanghai, China). The pEGFP-C1-ERα plasmid was purchased from Addgene (Cambridge, MA, USA), and pcDNA3-BMI1 plasmid was a gift from Dr. MH Yang (Institute of Clinical Medicine, National Yang-Ming University, Taipei city, Taiwan) [20]. A wild type *BMI1* promoter (pXP2-BMI1-Luc1000) from −1023 to −1 was constructed and a mutant *BMI1* promoter was generated by replacing a TGACC (−178–174) sequence in the wild type *BMI1* promoter with a GACCC sequence. The following antibodies were used in this study: anti-E-cadherin (DAKO, Glostrup, Denmark; NCH-38), anti-Bmi1 (Abcam, Cambridge, UK; ab126783), anti-ERα (Santa Cruz Biotechnology, CA, USA; sc-543), anti-β-actin (Santa Cruz Biotechnology, Dallas, TX, USA; sc-4777), PE-conjugated anti-CD24 (BD Biosciences, Bedford, MA, USA; ML5), APC-conjugated anti-CD44 (BD Pharmigen, San Jose, CA, USA; G44-26), PE-conjugated mouse IgG (BD; G155-178), and APC-conjugated mouse IgG (BD; 27–35).

### Transfections

We transfected 5 μg of the vectors, pEGFP-C1 or pEGFP-C1-ERα, containing full-length human ERα, into BT549 cells growing in 10-cm tissue culture plates using Lipofectamine 2000 (Invitrogen, Carlsbad, CA, USA). Stable clones were selected and established by culturing in Dulbecco's modified Eagle's medium (DMEM) plus 10% fetal bovine serum (FBS) containing 0.8 mg/mL G418 (Merck & Co., Whitehouse Station, NJ, USA). To clarify the role of Bmi1, 5 μg of pcDNA3 or pcDNA3-BMI1 was transfected into pEGFP-C1-ERα stable cells growing in 10-cm tissue culture plates for 48 h. ERα siRNA and scrambled siRNA were synthesized by Genepharma Biotech (Shanghai, China). ERα siRNA-1 sequences were as follows [2]: 5′-CGAGUAUGAUCCUACCAGAII-3′ (sense) and 5′-UCUGGUAGGAUCAUACUCGGA-3′ (antisense); siRNA-2 sequences: 5′-AAGCUACUGUUUGCUCCUAACTT-3′ (sense) and 5′-GUUAGGAGCAAACAGUAGCUUTT-3′ (antisense). Scrambled siRNA sequences were 5′-UUCUCCCGAACGUUCACGU-3′ (sense) and 5′-ACGUGACACGUUCGGAGAA-3′ (antisense). Transfection of siRNA involved the use of Lipofectamine 2000. Briefly, 5 or 50 nmol/L ERα siRNA or scrambled siRNA was transfected into cultured T47D cells (at 50% confluence) for 48 h.

### RNA isolation and RT-PCR

TRIzol reagent (Invitrogen, Carlsbad, CA) was used to isolate total RNA from cultured cells or fresh-frozen breast cancer tissue. The PrimeScript RT Reagent Kit (Takara, Dalian, China) with random primers was used to synthesize cDNA. PCR reactions involved 2 μL cDNA and the ABI PRISM 7300 Sequence Detection System (Applied Biosystems, Foster City, CA, USA). Expression was normalized to that of the β-actin housekeeping gene as an internal control. The primer sequences were for β-actin, 5′-AGCGAGCATCCCCCAAAGTT-3′ (sense) and 5′-GGGCACGAAGGCTCATCATT-3′ (antisense); ERα, 5′-TGCTTCAGGCTACCATTATGGA-3′ (sense) and 5′-TGGCTGGACACATATAGTCGTT-3′ (antisense); Bmi1, 5′-GCTGCCAATGGCTCTAATGAA-3′ (sense); and 5′-TGCTGGGCATCGTAAGTATCTT-3′ (antisense); E-cadherin, 5′-AAAGGCCCATTTCCTAAAAACCT-3′ (sense) and 5′-TGCGTTCTCTATCCAGAGGCT-3′.

### Chromatin immunoprecipitation (ChIP) assay

ChIP assays were performed as previously described [36]. In brief, T47D cells at 80% to 90% confluence growing in 10-cm dishes were treated with 1% formaldehyde for 10 min to cross-link proteins to DNA, and then sonicated four times for 10 sec by use of a sonicator with a microtip in a 1.5-mL tube. The resultant lysate underwent immunoprecipitation (IP) with 1 μg polyclonal anti-ERα antibody. Normal IgG was used as an IP control, and the supernatant was used as an input control. Immunoprecipitated complexes were collected by adding protein A/G-agarose/salmon sperm DNA beads and incubating samples for 2 h at 4ºC. The beads were then treated with RNase A (50 μg/mL) and proteinase K. DNA was extracted with phenol/chloroform and co-precipitated with glycogen, dissolved in 25 μL TE buffer, and subjected to PCR amplification for ERα binding sites in the BMI1 promoter using specific primers: BMI1-1, 5′-CGGGCCTGACTACACCGACAC-3′ (sense), and 5′-GGAAACTGACACCGGCTCCAA-3′ (antisense); BMI1-2, GCAGAGGAAAACCAGAAACG-3′ (sense) and 5′-TGGGCAGTATCTTTCCCTCTT-3′ (antisense). The acquired DNA was resolved on a 2% agarose gel and stained with Goldview.

### Electrophoretic mobility shift assay (EMSA)

Oligonucleotides (5′-AGCACGTGACCCGCTGGG-3′) containing putative binding sequences of ERα were labeled at the 3′-end with biotin and incubated with nuclear extracts harvested from BT549 cells transfected with pEGFP-C1-ERα. The binding reaction (20 min at room temperature) was carried out in a final volume of 20 μL containing the biotin-labeled probe (20 fmol), with nuclear extract according to the LightShift Chemiluminescent EMSA Kit (Beyotime Biotech, Haimen, China) manufacturer's protocol. In supershift experiments, the nuclear extract was preincubated with anti-ERα antibody for 30 min on ice. In non-specific binding assays, poly (dI:dC), nonspecific antibodies, including normal IgG or anti-Twist1 antibody, were added instead of anti-ERα antibody. In the competition assay, excess amounts of unlabeled competitors, including putative binding sequences and mutant sequences (5′-AGCACGGACCCCGCTGGG-3′), were added before the labeled probes. After binding, the samples were separated on 6% non-denaturing polyacrylamide gels and visualized by enhanced chemiluminescence.

### Cell migration and invasion assays

A modified cell migration assay was performed using BT549 cells stably transfected with pEGFP-C1 or pEGFP-C1-ERα cells as described previously [26]. An 8-μm pore-size Boyden chamber (BD) was used for *in vitro* migration and invasion assays. Cells (5 × 10^4^) incubated in DMEM supplemented with 1% bovine serum albumin were plated in the upper chamber, and 10% fetal bovine serum was added to DMEM in the lower chamber as a chemoattractant. A BD BioCoat Matrigel Invasion Chamber (BD) was used for the invasion assay. After 24 h (migration assay) or 72 h (invasion assay), cells on the upper side of the filter were removed, and cells that remained adherent to the underside of membrane were fixed in 4% formaldehyde and stained with crystal violet dye. Five random fields per membrane were photographed with the use of a BX51 microscope (Olympus, Tokyo, Japan) at ×400 magnification. The cells were counted and the mean number of the five fields was calculated to obtain a representative number of cells that had migrated/invaded across the membrane. Three independent experiments were performed for each assay.

### Wound healing assay

BT549 cells stably transfected with pEGFP-C1 or pEGFP-C1-ERα were seeded in 6-well plates at a density of 3 × 10^5^ cells/well. Cells were then serum-starved for 24 h, and a linear wound was created in the confluent monolayer by use of a pipette tip. Wound healing was photographed at 24 h intervals. Experiments were done in triplicate, and three random fields of each well were recorded.

### Flow-cytometric analysis of CD44 and CD24 expression

To analyze CD44 and CD24 expression, cells were trypsinized and suspended in phosphate buffered saline (PBS), plus 1% fetal bovine serum, at a density of 10^6^ cells/100 μL. Cells were incubated at 4°C in the dark for 40 min with PE-conjugated anti-CD44 antibody and APC-conjugated anti-CD24 antibody, or their respective isotype controls, at concentrations recommended by the manufacturer. Labeled cells were washed twice with PBS, then fixed in PBS containing 1% paraformaldehyde and analyzed with the use of a FACSCalibur flow cytometer (BD).

### Mammosphere assay

Single cells were plated at a density of 1,000 cells/mL in 96-well ultralow attachment plates (Corning, New York, USA). Cells were grown in serum-free DMEM/F12 supplemented with B27 (1:50, Invitrogen), 20 ng/mL endothelial growth factor, 20 ng/mL basic fibroblast growth factor and 4 μg/mL heparin (Sigma, St. Louis, MO, USA). Cells were cultured for 7 d, and mammospheres with >50 μm diameter were counted.

### Immunohistochemistry (IHC) of human breast cancer tissues

Human breast cancer specimens were obtained from 58 patients who underwent breast cancer surgery at the Cancer Hospital of Shantou University Medical College, China between 2010 and 2011. Written informed consent was obtained from each patient, and the study was approved by the Hospital Research Ethics Committee.

Serial formalin-fixed and paraffin-embedded tissues were sectioned at a 4 μm thickness, deparaffinized, and rehydrated in gradients of high percentage ethanol to distilled water. For quenching endogenous peroxidase activity, sections were immersed in 3% hydrogen peroxide for 15 min at room temperature. Antigen retrieval involved boiling in 10 mM sodium citrate buffer (pH 6) for 3 min in a pressure cooker, followed by cooling to room temperature. Sections were then incubated with the primary antibody at 4ºC overnight, washed three times in PBS for 5 min, and incubated with horseradish peroxidase-conjugated goat anti-mouse/rabbit IgG antibody (ZSGB-Bio, Beijing, China) at room temperature for 30 min, followed by 3, 3′-diaminobenzidine tetra-hydrochloride (DAB) staining. Sections were lightly counterstained with hematoxylin.

The expression of ERα, Bmi1 and E-cadherin was detected using specific antibodies, with normal mouse or rabbit IgG (Santa Cruz Biotechnology) used as the negative control. Cases were considered as positive for ERα if nuclear immunoreactivity was present in ≥10% of tumor cells. For nuclear Bmi1 staining, the percentage of stained cells was categorized into 0 to 3+ as previously described [20]: 0, no staining; 1+, 1–25%; 2+, 25–50%; and 3+, > 50% nuclear staining. Only 3+ was considered as a positive IHC result. E-cadherin expression was interpreted as either normal (strong) or aberrant (reduced or absent). Aberrant staining was defined as either negative staining or <50% membranous staining of the population of cells examined. Normal staining was defined as ≥50% membranous staining of the cancer cells [37]. The percent of positively stained cells was evaluated, in the same section of tissue, to analyze the correlation between Bmi1 and ERα or E-cadherin.

### *In vivo* metastasis in mice

Eight- to 10-week-old female BALB/C nude mice were intravenously injected, via the tail vein, with 5 × 10^7^ pEGFP-C1 or pEGFP-C1-ERα stable cells in 0.1 mL PBS (5 mice for each group). Six weeks after injection, mice were euthanized to grossly detect metastases. The visceral organs, such as lung and liver, were fixed with formalin, then paraffin-embedded and stained with hematoxylin and eosin (H&E). Metastatic lesions were evaluated by microscopic examination. The animal study was approved by the Institutional Animal Care and Use Committee of Shantou University Medical College.

### Statistical analysis

Statistical analysis was performed using SPSS 16.0 (SPSS Inc., Chicago, IL, USA). Differences among variables were assessed by χ2 analysis, Spearman's Rank Correlation Test or 2-tailed Student's *t* tests. Data were presented as the mean ± SEM unless otherwise indicated. Two-sided *P* < 0.05 was considered statistically significant. Each experiment was performed at least three times.

## SUPPLEMENTARY FIGURES



## References

[1] Guttilla IK, Adams BD, White BA (2012). ERalpha, microRNAs, and the epithelial-mesenchymal transition in breast cancer. Trends in endocrinology and metabolism: TEM.

[2] Ye Y, Xiao Y, Wang W, Yearsley K, Gao JX, Barsky SH (2008). ERalpha suppresses slug expression directly by transcriptional repression. The Biochemical journal.

[3] Parl FF, Schmidt BP, Dupont WD, Wagner RK (1984). Prognostic significance of estrogen receptor status in breast cancer in relation to tumor stage, axillary node metastasis, and histopathologic grading. Cancer.

[4] Shen HM, Tergaonkar V (2009). NFkappaB signaling in carcinogenesis and as a potential molecular target for cancer therapy. Apoptosis : an international journal on programmed cell death.

[5] Taylor MA, Parvani JG, Schiemann WP (2010). The pathophysiology of epithelial-mesenchymal transition induced by transforming growth factor-beta in normal and malignant mammary epithelial cells. Journal of mammary gland biology and neoplasia.

[6] Wang X, Belguise K, Kersual N, Kirsch KH, Mineva ND, Galtier F, Chalbos D, Sonenshein GE (2007). Oestrogen signalling inhibits invasive phenotype by repressing RelB and its target BCL2. Nature cell biology.

[7] Ye Y, Xiao Y, Wang W, Yearsley K, Gao JX, Shetuni B, Barsky SH (2010). ERalpha signaling through slug regulates E-cadherin and EMT. Oncogene.

[8] Chaffer CL, Brueckmann I, Scheel C, Kaestli AJ, Wiggins PA, Rodrigues LO, Brooks M, Reinhardt F, Su Y, Polyak K, Arendt LM, Kuperwasser C, Bierie B, Weinberg RA (2011). Normal and neoplastic nonstem cells can spontaneously convert to a stem-like state. Proceedings of the National Academy of Sciences of the United States of America.

[9] Peppercorn J, Perou CM, Carey LA (2008). Molecular subtypes in breast cancer evaluation and management: divide and conquer. Cancer investigation.

[10] May CD, Sphyris N, Evans KW, Werden SJ, Guo W, Mani SA (2011). Epithelial-mesenchymal transition and cancer stem cells: a dangerously dynamic duo in breast cancer progression. Breast cancer research : BCR.

[11] Singh A, Settleman J (2010). EMT, cancer stem cells and drug resistance: an emerging axis of evil in the war on cancer. Oncogene.

[12] Raimondi C, Gianni W, Cortesi E, Gazzaniga P (2010). Cancer stem cells and epithelial-mesenchymal transition: revisiting minimal residual disease. Current cancer drug targets.

[13] Thiery JP, Acloque H, Huang RY, Nieto MA (2009). Epithelial-mesenchymal transitions in development and disease. Cell.

[14] Cao L, Bombard J, Cintron K, Sheedy J, Weetall ML, Davis TW (2011). BMI1 as a novel target for drug discovery in cancer. Journal of cellular biochemistry.

[15] Huber GF, Albinger-Hegyi A, Soltermann A, Roessle M, Graf N, Haerle SK, Holzmann D, Moch H, Hegyi I (2011). Expression patterns of Bmi-1 and p16 significantly correlate with overall, disease-specific, and recurrence-free survival in oropharyngeal squamous cell carcinoma. Cancer.

[16] Park IK, Morrison SJ, Clarke MF (2004). Bmi1, stem cells, and senescence regulation. The Journal of clinical investigation.

[17] Cui H, Hu B, Li T, Ma J, Alam G, Gunning WT, Ding HF (2007). Bmi-1 is essential for the tumorigenicity of neuroblastoma cells. The American journal of pathology.

[18] Mihic-Probst D, Kuster A, Kilgus S, Bode-Lesniewska B, Ingold-Heppner B, Leung C, Storz M, Seifert B, Marino S, Schraml P, Dummer R, Moch H (2007). Consistent expression of the stem cell renewal factor BMI-1 in primary and metastatic melanoma. International journal of cancer Journal international du cancer.

[19] Liu S, Dontu G, Mantle ID, Patel S, Ahn NS, Jackson KW, Suri P, Wicha MS (2006). Hedgehog signaling and Bmi-1 regulate self-renewal of normal and malignant human mammary stem cells. Cancer research.

[20] Yang MH, Hsu DS, Wang HW, Wang HJ, Lan HY, Yang WH, Huang CH, Kao SY, Tzeng CH, Tai SK, Chang SY, Lee OK, Wu KJ (2010). Bmi1 is essential in Twist1-induced epithelial-mesenchymal transition. Nature cell biology.

[21] Song LB, Li J, Liao WT, Feng Y, Yu CP, Hu LJ, Kong QL, Xu LH, Zhang X, Liu WL, Li MZ, Zhang L, Kang TB, Fu LW, Huang WL, Xia YF (2009). The polycomb group protein Bmi-1 represses the tumor suppressor PTEN and induces epithelial-mesenchymal transition in human nasopharyngeal epithelial cells. The Journal of clinical investigation.

[22] Wellner U, Schubert J, Burk UC, Schmalhofer O, Zhu F, Sonntag A, Waldvogel B, Vannier C, Darling D, zur Hausen A, Brunton VG, Morton J, Sansom O, Schuler J, Stemmler MP, Herzberger C (2009). The EMT-activator ZEB1 promotes tumorigenicity by repressing stemness-inhibiting microRNAs. Nature cell biology.

[23] Fujita N, Jaye DL, Kajita M, Geigerman C, Moreno CS, Wade PA (2003). MTA3, a Mi-2/NuRD complex subunit, regulates an invasive growth pathway in breast cancer. Cell.

[24] Moggs JG, Murphy TC, Lim FL, Moore DJ, Stuckey R, Antrobus K, Kimber I, Orphanides G (2005). Anti-proliferative effect of estrogen in breast cancer cells that re-express ERalpha is mediated by aberrant regulation of cell cycle genes. Journal of molecular endocrinology.

[25] Batlle E, Sancho E, Franci C, Dominguez D, Monfar M, Baulida J, Garcia De Herreros A (2000). The transcription factor snail is a repressor of E-cadherin gene expression in epithelial tumour cells. Nature cell biology.

[26] Comijn J, Berx G, Vermassen P, Verschueren K, van Grunsven L, Bruyneel E, Mareel M, Huylebroeck D, van Roy F (2001). The two-handed E box binding zinc finger protein SIP1 downregulates E-cadherin and induces invasion. Molecular cell.

[27] Cano A, Perez-Moreno MA, Rodrigo I, Locascio A, Blanco MJ, del Barrio MG, Portillo F, Nieto MA (2000). The transcription factor snail controls epithelial-mesenchymal transitions by repressing E-cadherin expression. Nature cell biology.

[28] Bolos V, Peinado H, Perez-Moreno MA, Fraga MF, Esteller M, Cano A (2003). The transcription factor Slug represses E-cadherin expression and induces epithelial to mesenchymal transitions: a comparison with Snail and E47 repressors. Journal of cell science.

[29] Yang JY, Zong CS, Xia W, Wei Y, Ali-Seyed M, Li Z, Broglio K, Berry DA, Hung MC (2006). MDM2 promotes cell motility and invasiveness by regulating E-cadherin degradation. Molecular and cellular biology.

[30] Sobrado VR, Moreno-Bueno G, Cubillo E, Holt LJ, Nieto MA, Portillo F, Cano A (2009). The class I bHLH factors E2-2A and E2-2B regulate EMT. Journal of cell science.

[31] Wong AS, Gumbiner BM (2003). Adhesion-independent mechanism for suppression of tumor cell invasion by E-cadherin. The Journal of cell biology.

[32] Guo BH, Feng Y, Zhang R, Xu LH, Li MZ, Kung HF, Song LB, Zeng MS (2011). Bmi-1 promotes invasion and metastasis, and its elevated expression is correlated with an advanced stage of breast cancer. Molecular cancer.

[33] Beato M, Herrlich P, Schutz G (1995). Steroid hormone receptors: many actors in search of a plot. Cell.

[34] Petz LN, Nardulli AM (2000). Sp1 binding sites and an estrogen response element half-site are involved in regulation of the human progesterone receptor A promoter. Molecular endocrinology.

[35] Wang H, Liu H, Li X, Zhao J, Zhang H, Mao J, Zou Y, Zhang H, Zhang S, Hou W, Hou L, McNutt MA, Zhang B (2014). Estrogen receptor alpha-coupled Bmi1 regulation pathway in breast cancer and its clinical implications. BMC cancer.

[36] Zhang D, Xie X, Chen Y, Hammock BD, Kong W, Zhu Y (2012). Homocysteine upregulates soluble epoxide hydrolase in vascular endothelium *in vitro* and *in vivo*. Circulation research.

[37] Yang MH, Chang SY, Chiou SH, Liu CJ, Chi CW, Chen PM, Teng SC, Wu KJ (2007). Overexpression of NBS1 induces epithelial-mesenchymal transition and co-expression of NBS1 and Snail predicts metastasis of head and neck cancer. Oncogene.

