# Supplementary material for: ERα inhibits epithelial-mesenchymal transition by suppressing Bmi1 in breast cancer
Source: Oncotarget. 2015 May 13;6(25):21704–17. doi: 10.18632/oncotarget.3966 (PMC4673297; doi:10.18632/oncotarget.3966)
Supplement: Supplementary file 1 [file oncotarget-06-21704-s001.pdf]

SUPPLEMENTARY FIGURES

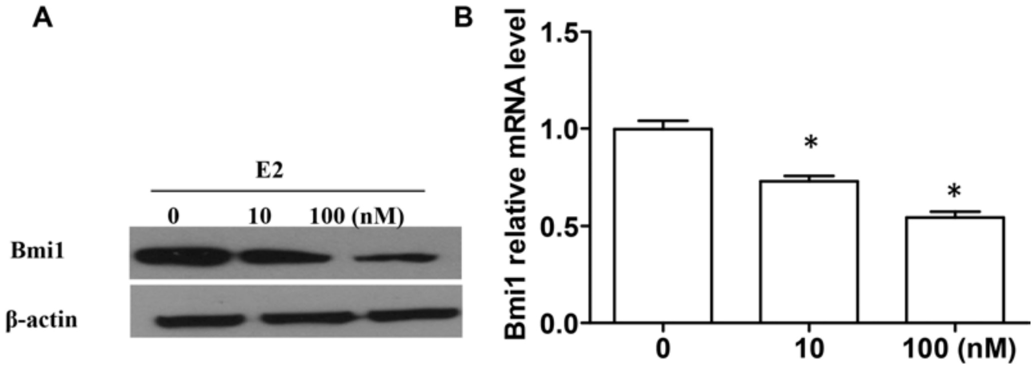

Supplementary Figure S1: E2 downregulates Bmi1 expression in T47D cells. **A–B.** Western blot and quantitative real-time RT-PCR analysis of protein and mRNA expression of Bmi1 in T47D cells after E2 treatment for 24 h. T47D cells were maintained in phenol red-free DMEM with 10% dextran-coated charcoal-treated FBS for 48 h, and cells were then treated with either ethanol vehicle or E2 (10 or 100 nM) for 24 h. Cells were harvested and analyzed for Bmi1 mRNA and protein levels. *$P < 0.05$ compared with ethanol vehicle (Student's *t* test).

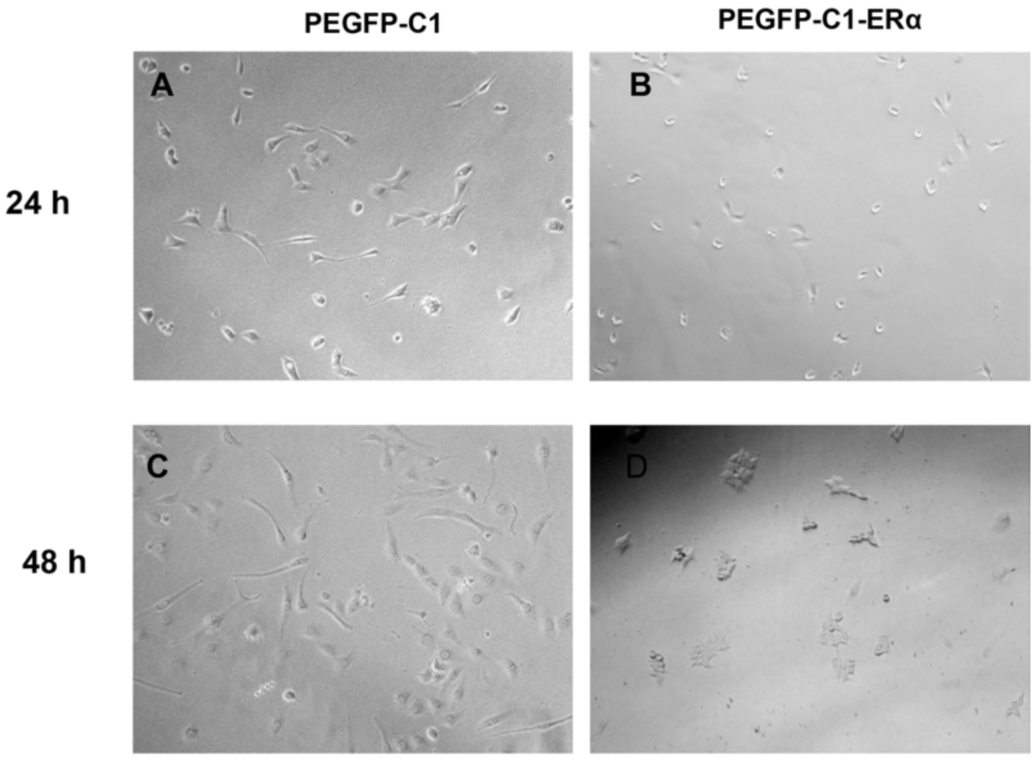

Supplementary Figure S2: Morphology of pEGFP-C1 and pEGFP-C1-ERα BT549 cells. Morphology of pEGFP-C1 and pEGFP-C1-ERα BT549 cells after 24 and 48 h culture. Upper: morphology observed after 24 h culture. Lower: morphology observed after 48 h culture. pEGFP-C1: pEGFP-C1 BT549 stable cells; pEGFP-C1-ERα: pEGFP-C1-ERα BT549 stable cells. Magnification, 400×.

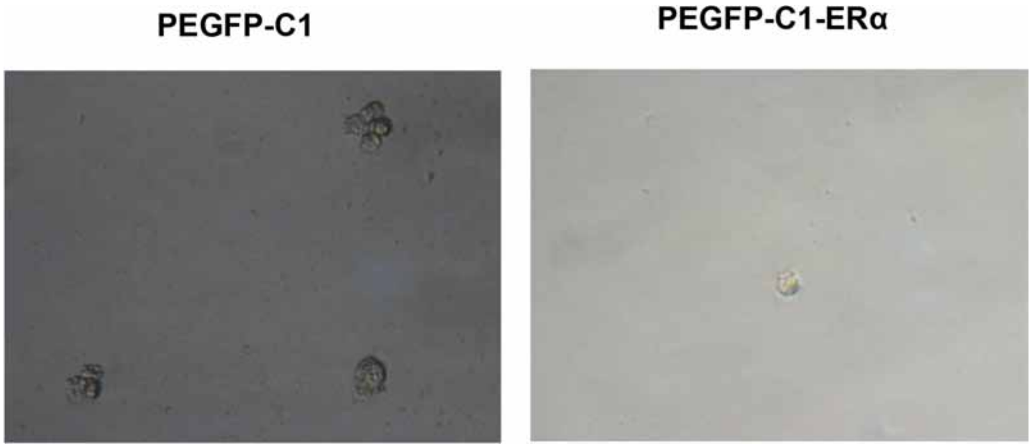

Supplementary Figure S3: Representative images of mammosphere forming units (MFUs) observed in pEGFP-C1 and pEGFP-C1-ERα BT549 cells under low magnification. pEGFP-C1: pEGFP-C1 BT549 stable cells; pEGFP-C1-ERα: pEGFP-C1-ERα BT549 stable cells. Magnification, 200×.
